# Simulated vertical electric field data: An estimation from an improved coupling model for the lithosphere-atmosphere-ionosphere system

**DOI:** 10.1016/j.dib.2019.104513

**Published:** 2019-09-17

**Authors:** Boris E. Prokhorov, Oleg V. Zolotov, Maria A. Knyazeva, Yulia V. Romanovskaya

**Affiliations:** a Helmholtz Center Potsdam, GFZ German Research Center for Geosciences, Potsdam, Germany; b Near-Earth Environment Computer Modeling Laboratory, Murmansk Arctic State University, Murmansk, Russia; c Chair of Mathematics, Information Systems and Software Engineering, Murmansk State Technical University, Murmansk, Russia

**Keywords:** Electric fields, Seismogenic electric currents, Simulations

## Abstract

Researches in the field of the lithosphere-atmosphere-ionosphere (LAI) coupling involve discussions on the physical mechanisms that might be responsible for that coupling. Hypotheses that are based on an electromagnetic physical mechanism of seismo-ionosphere disturbances generation trigger discussions on the required electric fields. Kuo and Lee [doi: 10.1002/2016JA023441] proposed and discussed an improved coupling model for the lithosphere-atmosphere-ionosphere system but did not provide any electric fields values (*E*_*z*_ tables, figures or datasets). In this paper we fill this shortage and present corresponding numerical estimations (dataset) of the required vertical electric fields values *E*_*z*_. Our dataset is valuable for comparison of the LAI-models with the electric fields observations and to contrast different LAI-coupling models with each other.

**Table:** Specification table

Subject area	Lithosphere-atmosphere-ionosphere coupling
More specific subject area	Electric fields preceding strong seismic events
Type of data	*E*_*z*_ 3D distributions in binary NumPy.npz file
How data was acquired	The dataset (vertical electric fields) was estimated from simulated seismogenic electric currents data via the Ohm's law
Data format	Raw, modelled
Experimental factors	Vertical component of the electric fields 3D distribution possibly associated with pre-earthquakes’ phenomena.
Experimental features	Vertical electric fields *E*_*z*_ dataset is derived from vertical electric currents data provided with an improved coupling model for the lithosphere-atmosphere-ionosphere system using the same spatial domain and numerical grid
Data source location	Murmansk Arctic State University, Murmansk, Russia
Data accessibility	The data have been made available online via public repository at https://gitlab.com/zolotov/dib/tree/master/dib2019
Related research article	B.E. Prokhorov, O.V. Zolotov, Comments on “An improved coupling model for the lithosphere-atmosphere-ionosphere system” by Kuo et al. [2014], J. Geophys. Res. Space Physics, 122, 4865–4868, https://doi.org/10.1002/2016JA023441[1].

**Table:** 

**Value of the data** • This dataset is valuable to validate the specific electromagnetic model of the lithosphere-atmosphere-ionosphere (LAI)-coupling in contrast with the observations of pre-seismic and regular ground-to-ionosphere vertical electric fields. • This dataset is valuable to cross-compare features of an empirical, semi-empirical, and self-consistent electromagnetic LAI-coupling models. • The dataset may reveal its place during planning of the *in-situ* electric fields measurements to validate the specific LAI-coupling models and for experimenting with multi-parameter earthquake detection and warning systems.

## 1 Data

The proposed dataset contains numerically evaluated 3D distributions of the vertical electric fields *E*_*z*_ (in units of V/m) that are attributed to the simulated by Kuo and Lee [2] seismogenic electric currents floating between the Earth's surface (i.e., the ground) and the ionosphere. In our dataset the spatial data domain and numerical grid are chosen the same as in paper [2]: 1000 km … 1000 km along the X axis (201 grid nodes), −1000 km … 1000 km along the Y axis (201 grid nodes), and 0 … 130 km along the Z axis (132 grid nodes), i.e. the altitude over the Earth's surface. Zero (0 km) altitude corresponds to the Earth's surface (i.e., the lower boundary), and 130 km corresponds to the upper boundary of the domain. The dataset NumPy npz-file contains x-coordinates (labeled as ‘x’), y-coordinates (labeled as ‘y’), z-coordinates (labeled as ‘altitudes’), and 3D *E*_*z*_ spatial distribution (labeled as ‘ez’).

**Fig. 1:**
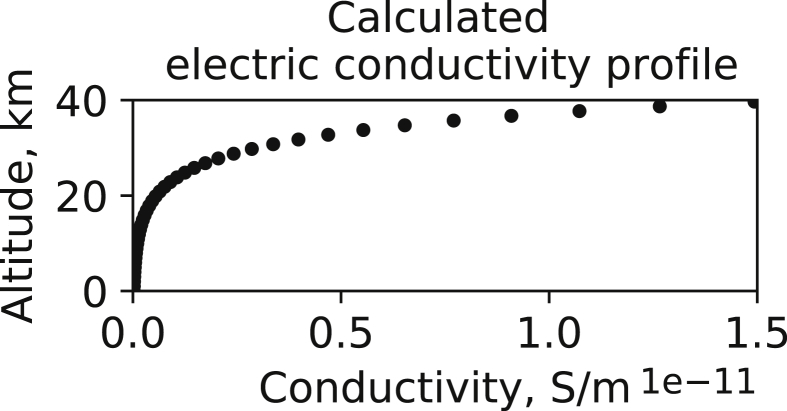
Altitudinal variation of the electric conductivity (S/m) profile calculated for the considered grid nodes. Each value on the plot is represented with a filled dot. X-axis values are scaled by the factor 10^−11^, thus, label 1.5 denotes 1.5∙10^−11^ S/m.

**Fig. 2:**
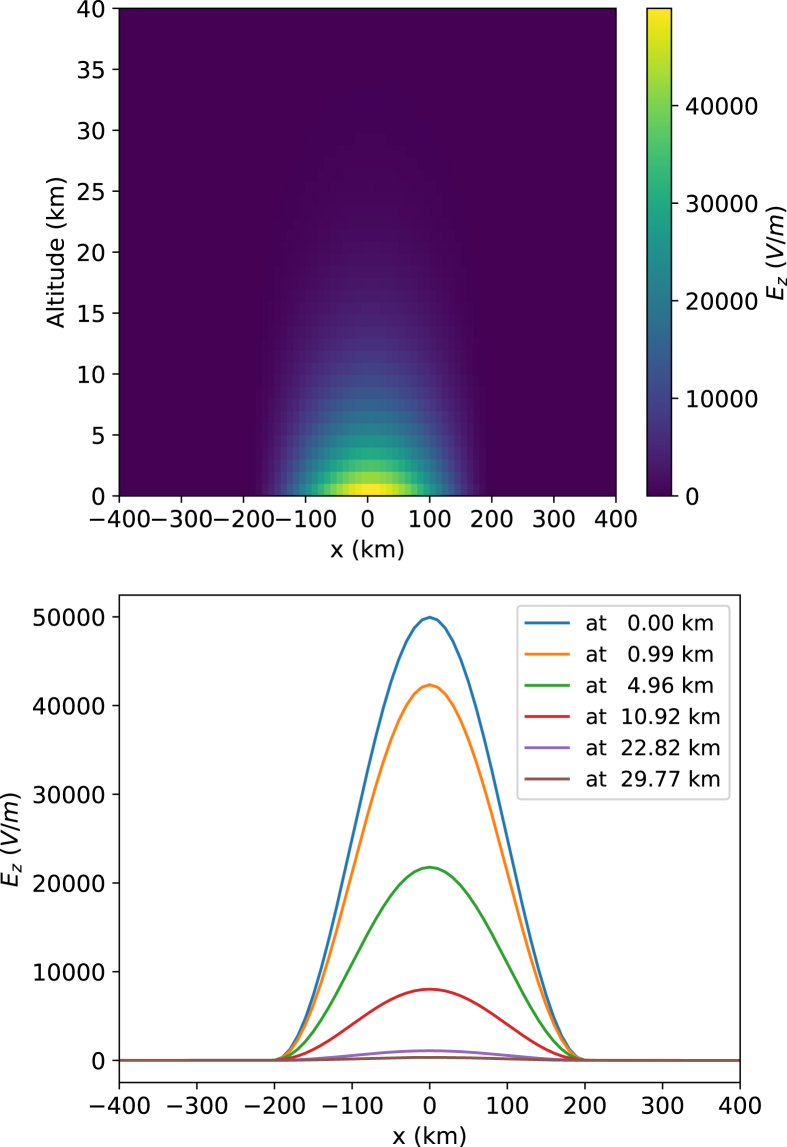
Illustration of vertical electric fields *E*_*z*_ (V/m) spatial distributions. Top panel presents the map of the vertical electric fields *E*_*z*_ distribution in the X-Z plane (cross-section for the y = 0 km). Bottom panel presents vertical electric fields *E*_*z*_ variations along the x axis at the specified altitudes (i.e., z-values, see the legend) in the given above X-Z plane.

The proposed dataset is a modelled dataset that is not linked to any specific Earth's locations. Fig. 1 displays altitudinal variation of the electric conductivity profile used to recalculate vertical electric fields *E*_*z*_ data. Required seismogenic electric currents data are taken according to Ref. [3]. Fig. 2 describes corresponding *E*_*z*_ variations for a characteristic cross-section plane (top panel) and at specific altitudes (bottom plane).

## 2 Experimental design, materials, and methods

Unresolved strong earthquakes prediction problem triggered multiple interdisciplinary attempts to build self-consistent models that describe lithosphere-atmosphere-ionosphere (LAI) coupling. Those relying on an electromagnetic LAI-coupling usually require either (1) significantly enhanced electric conductivity values near the ground at the bottom part of the electric conductivity profile or (2) very intense electric field. To consistently explain the items above or to provide a complete qualitative model that does not rely on significant electric conductivity or electric fields modifications is a challenge nowadays for the electromagnetic LAI-coupling (see a discussion in, e.g. Ref. [1], vs [2]). It is a must for understanding of the underlying physical processes and to assess the possibility of the LAI-based strong earthquakes' forecasting. To validate an electromagnetic LAI-coupling model, it is important to have the electric conductivity, electric fields and electric currents data simultaneously to be able to contrast all of them with available observations or reference data. Among the named pre-earthquake ground-to-ionosphere *in-situ* seismogenic electric currents are the worst known ones. The representation of those data is expected to facilitate the analysis and is desirable to be unambiguous and explicit.

Unfortunately, researchers [2] provide nothing on the electric fields data (i.e., any discussion or *E*_*z*_ values, tables or figures, etc.) that correspond to their simulated data on ground-to-ionosphere electric currents of seismic origin. In the supporting information file only [4] they state that “due to limit of max data size 100M on website, the grid numbers in Figure 3 are reduced by half. Since the conductivity modeling the atmosphere is given analytically, the electric fields can be easily obtained from the current data with the Ohm's law”. Here we note, that a few previous papers by Kuo and coauthors [5], [6] suffered from a lack of formulations or data required to reproduce their results quantitatively. Despite the denoted ease of *E*_*z*_ data recalculation, paper [2] requires the reader to perform extra efforts to obtain estimates of corresponding *E*_*z*_ values. We must note that *E*_*z*_ data are among the first ones to validate when deal with electromagnetic LAI-coupling models. A validation usually includes *E*_*z*_ data comparison with a few known reference entities (so called “fair-weather” electric fields, thunderstorm electric fields, derived in laboratory experiments electric fields, possibly, a few *in-situ* measurements, etc.). A contrast with other electromagnetic LAI-coupling models often reveal principal similarities and divergences, thus, allowing to highlight model's advances and to reveal principle challenges to solve.

To facilitate the aforementioned opportunities, we re-calculate vertical electric fields from (1) the original electric currents data [3] and (2) the analytical formulation for the electric conductivity variation. To avoid any interpolation errors and to allow direct intercomparison of our data, we use the same spatial domain and numerical grid as in the original electric currents dataset.

In this paper we utilize as input the dataset [3] which is originally provided in the proprietary MATLAB file format. According to the description [4] it includes three numerically simulated components *j*_*z*_, *j*_*y*_, *j*_*z*_ of electric currents densities (in units of A/km^2^), position (x, y and z in units of km) and corresponding grid number (nx, ny and nz).

To calculate vertical electric field values *E*_*z*_, we utilize the following analytical formulation of the electric conductivity profile according to the equation 8 in paper [2]:

(1) $$\sigma \text{(z) }=\;\sigma_{\text{0}}\;\text{exp(z/}h_{\text{z}}\text{),}$$

where σ_0_ is the conductivity at z = 0 km (i.e., on the ground), σ_0_ = 2∙10^−14^ S/m, and h_z_ = 6 km is the conductivity scale height.

It is clear that in the formulation 1 the electric conductivity σ is a scalar function. It is also clear that the given conductivity formula does not depend on the x or y co-ordinates. Corresponding altitudinal variation of the electric conductivity profile is illustrated in Fig. 1.

To calculate the vertical electric fields *E*_*z*_ dataset, we use the Ohm's law which, given the conductivity is a scalar and electric currents are curl-free, has the following form:

(2) $$J_{z}\;=\;\sigma \;E_{z}$$

where σ is the electric conductivity defined according to equation (1), and *J*_*z*_ is the vertical electric current density.

Described in this paper vertical electric fields *E*_*z*_ dataset is obtained according to the equation (2) where the electric conductivity is taken according to the equation (1) and vertical electric currents values are taken directly from the dataset [3]. Features of the vertical electric fields *E*_*z*_ spatial distribution are illustrated in Fig. 2.

We published the vertical electric fields *E*_*z*_ dataset and the source code as Jupyter (formerly known as IPython) notebooks at gitlab public repository (see URL in the specification table) to make our results reproducible by other researchers and to facilitate further analysis and discussions. To re-use the notebooks one will need Jupyter with Python3 and SciPy, NumPy, and matplotlib packages installed. It allows data investigation with opensource and free software without the need for a proprietary data formats and software preventing the so called “vendor lock-in”, thus, facilitating scientific collaboration and making the data easier to find, re-use and validate. Our data representation is suitable for use as input parameters to other models that require electric fields values in the considered spatial domain. It is also suitable for cross-comparison with other models' data and observations (e.g., “fair-weather” electric fields, thunderstorm electric fields, empirical, analytical or numerical estimations, etc.).

### 2.1 Limitations

To re-calculate ground-to-ionosphere vertical electric fields *E*_*z*_ we assume that all the following conditions are met:

- Electric currents are purely conductivity currents, i.e. they are due to the conduction only;
- All transitional processes have passed, i.e. the currents are steady-state currents;
- Vertical profile of the electric conductivity is a scalar function of an altitude.

Due to the above, it is reasonable to analyze the calculated this way vertical electric fields *E*_*z*_ data from the Earth's ground (0 km) up to the 40–50 km above the Earth's surface although the dataset upper boundary is 130 km to meet the data domain in Ref. [2]. It is not reasonable to analyze the proposed *E*_*z*_ data in the ionosphere (i.e. at altitudes >60–80 km) where the electric conductivity becomes a tensor.
